# Algorithm-Guided Experimentation for Optimization of High-Performance Perovskite Solar Cells

**DOI:** 10.1021/acsenergylett.5c02477

**Published:** 2025-11-10

**Authors:** Donghyun Oh, Sanggyun Kim, Carlo A. R. Perini, Juan-Pablo Correa-Baena, Nikolaos V. Sahinidis

**Affiliations:** † School of Chemical and Biomolecular Engineering, 1372 Georgia Institute of Technology, Atlanta, Georgia 30332, United States; ‡ School of Materials Science and Engineering, Georgia Institute of Technology, Atlanta, Georgia 30332, United States; § School of Chemistry and Biochemistry, Georgia Institute of Technology, Atlanta, Georgia 30332, United States; ∥ H. Milton Stewart School of Industrial and Systems Engineering, Georgia Institute of Technology, Atlanta, Georgia 30332, United States

## Abstract

Achieving high performance in perovskite solar cells (PSCs) is challenging due to the numerous complex variables involved across multiple fabrication steps. The strong interdependence among design variables, such as fabrication processing parameters and material composition, further complicates the search for optimal designs in a vast design space. In this work, we present a data-driven, algorithm-guided experimentation framework for the systematic optimization of PSC performance. We employed a model-based, derivative-free optimization algorithm to explore the design space of key processing parameters and identify promising regions for experimentation. By optimizing up to six processing parameters across the device structure and requiring fewer than 100 tested designs, we improved reverse-scanning power conversion efficiency from 20.3% to 23.1% without altering chemical ingredients or device configuration. These results demonstrate an effective combination of mathematical optimization and experimental research, which can benefit applications across disciplines that involve complex, interrelated variables requiring systematic optimization.

## Graphical abstract

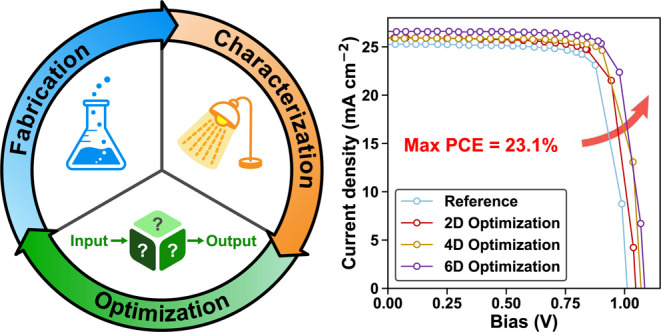

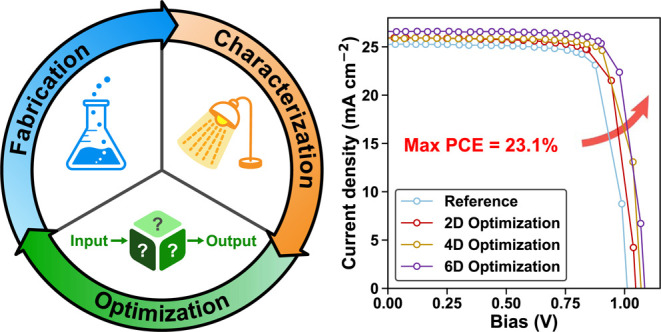

Perovskite solar cells (PSCs) have undergone remarkable advancements in performance in recent years.^1–6^ Experimental efforts in areas such as compositional engineering^7,8^ and defect passivation^9–13^ have largely driven this progress and continued to expand the design space of PSCs. Despite these breakthroughs, the systematic and rigorous optimization of numerous variables in PSC design and fabrication, many of which have a profound impact on performance, remains underexplored. In particular, understanding the impact of material composition and fabrication processing variables on device performance is intricate, and their interconnectedness further complicates the discovery of optimal designs in a vast design space. A major challenge in achieving high-performance PSCs stems from this complex interplay of design variables that are rarely optimized in a structured and comprehensive manner. Much of the optimization work in PSCs still relies heavily on manual exploration through trial-and-error and empirical decisions, navigating the vast and ever-growing design space without fully leveraging systematic optimization methods.

Recently, machine learning (ML) has gained significant attention in materials research for its predictive capacity and ability to accelerate material discovery and engineering. While the applications of ML in PSC systems have primarily utilized computational data to screen perovskite materials with desirable properties like synthesizability,^14–17^ suitable band gap,^15,18–27^ and stability,^20,26–30^ there is growing interest in utilizing experimental data,^31–34^ particularly for PSC device optimization.^35–54^ One approach is to compile pre-existing data on PSC performance from literature or open databases and train ML models for predictions or inverse device design.^35–37,40,42–50,52^ However, as experiments with PSCs often suffer from poor reproducibility across different research groups,^55–57^ ML models trained on such compiled data sets may face significant limitations in reliability. Alternatively, researchers have explored the use of in-house experimental data collected under a consistent laboratory environment to enhance the reliability of ML models.^38,39,41,51,53,54^ Nevertheless, the task of collecting sufficient data on device performance to train complex ML models can far exceed the capacity of a single laboratory. Consequently, these models may still struggle with data scarcity, limiting their predictive accuracy and effectiveness in optimizing device performance.

Amid the challenges of data-driven approaches in high-performance PSC optimization, optimization algorithms can serve as a systematic approach for guiding data collection and identifying optimal designs without the demand of excessive data. For instance, MacLeod et al.^58^ employed Bayesian optimization (BO) to optimize the two-dimensional design space of annealing time and dopant concentration and maximized the hole mobility of hole transport material with 35 tested conditions. Liu et al.^59^ utilized a BO framework and achieved a power conversion efficiency (PCE) of 18.5% with 100 tested conditions by optimizing six processing variables in perovskite deposition via open-air rapid spray plasma processing technique. Xu et al.^60^ applied BO for optimizing four variables in the fabrication of flexible PSCs and achieved a PCE near 11.5% by testing 48 conditions. Zhang et al.^61^ used BO to optimize six processing variables involved in the spin-coating deposition of perovskite layers with 24 tested conditions and demonstrated an improved PCE of 21.6% superior to manual step-by-step optimization results. While BO has been the predominant choice of algorithm in most optimization efforts, it is known to face a considerable scalability issue as the number of degrees of freedom increases,^62^ which compromises its effectiveness and requires increasingly larger data collection. This difficulty is exacerbated by the rising computational cost of fitting global surrogate models, such as Gaussian processes that are commonly used for BO, along with the increasing complexity of solving the subproblem of optimizing the acquisition function to determine the next sample points as the number of data points grows. Most importantly, its application in PSC optimization has mainly been on either the perovskite material alone or an individual layer within the multilayered architecture, failing to address the complex interlayer interactions across the device stack.

In the broader scope of optimization, derivative-free optimization (DFO)^63^ can provide a systematic framework for guiding experimentation and finding optimal conditions efficiently. This class of data-driven optimization algorithms, which encompasses BO, aims to achieve optimization when the algebraic expressions of the objective, constraints, and their mathematical derivatives are unavailable or intractable to directly optimize. DFO has successfully addressed numerous real-world data-driven optimization problems across various disciplines.^64–73^ Many of the state-of-the-art DFO solvers,^74–76^ which have demonstrated superior performance over BO especially in high-dimensional optimization problems,^77^ have not yet been widely adopted for optimizing fully assembled PSC devices. Employing those algorithms offers a structured approach for optimizing the intricate relationships between processing variables, which may uncover novel designs and advance PSCs toward their full potential.

Herein, we report a successful use of DFO algorithm-guided experimentation that systematically optimized up to six processing variables across multiple layers of the PSC device stack. By testing fewer than 100 different processing conditions without altering chemical ingredients or device configuration, we achieved significant improvements in PCE of cesium formamidinium (FA) lead iodide (Cs_0.09_FA_0.91_PbI_3_) perovskite-based PSC from 20.3% of the reference to 23.1% of the best-performing design. The substantial progress and the resulting stable, efficient PSC design demonstrate the impact of our approach as a practical framework for researchers across disciplines aiming to optimize complex variables that are otherwise difficult to address manually.

## Algorithm-Guided Experimentation

Figure 1a presents a flowchart of the combined experimental-computational framework where we utilized a systematic DFO algorithm to guide the experimentation and discover novel designs of PSCs. We began with a PSC device configuration shown in Figure 1b, which was fabricated using pure Cs_0.09_FA_0.91_PbI_3_ perovskite without the inclusion of additives such as methylammonium chloride. We then selected several key design variables, including chemical concentration and processing variables involved in device fabrication, which are illustrated in Figure S1 and detailed in the Methods. These variables were readily and directly controllable, allowing us to observe their impact on output performance as we modified them. We defined a range for each variable and performed an initial sampling of the input space defined by the lower and upper bound of each variable we aimed to optimize. As such, the input space encompassed all possible device designs within the specified ranges of the design variables. This minimal initial sampling was designed to collect data across the search space and provide that information to the optimization algorithm.

**Figure 1:**
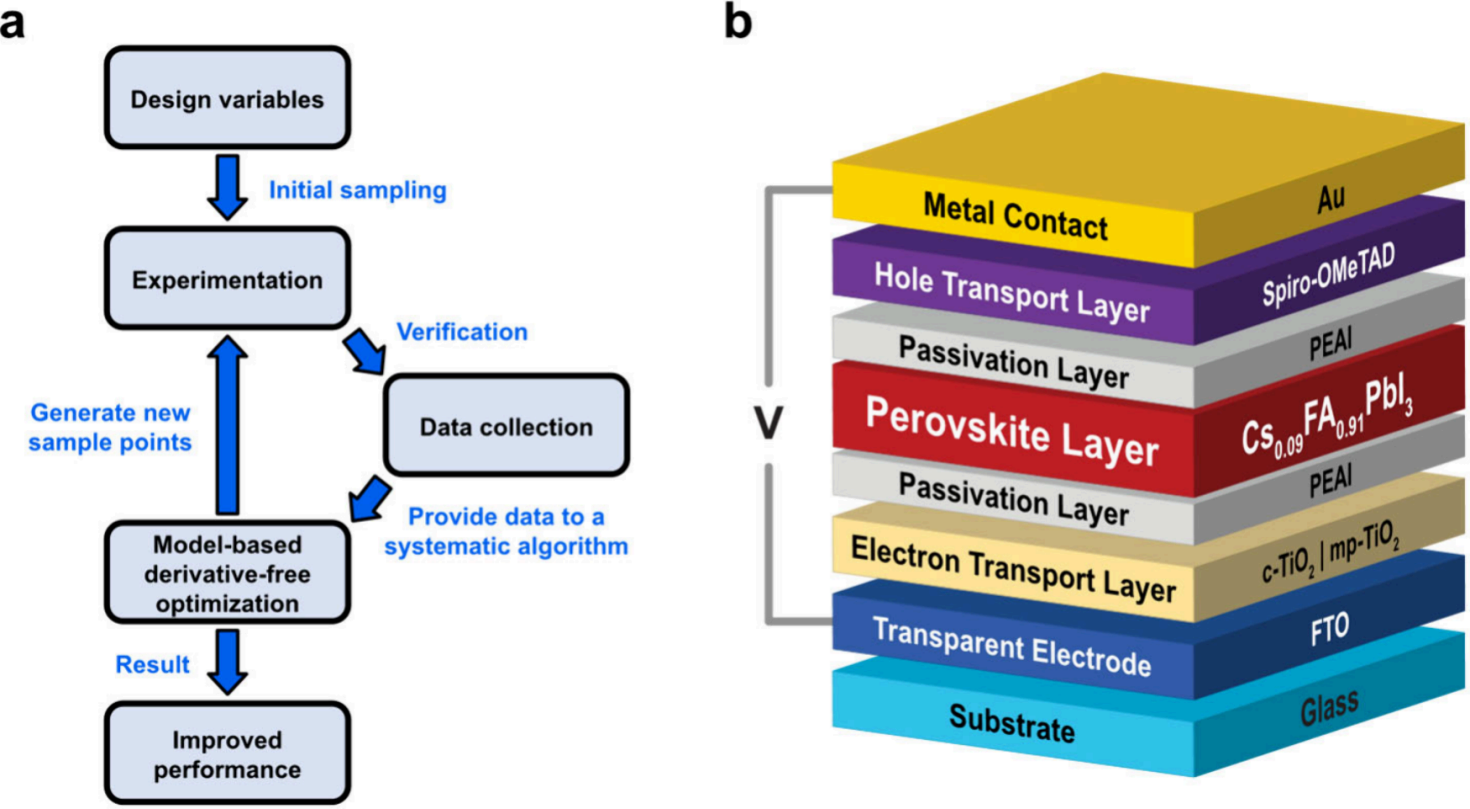
Algorithm-guided experimentation and PSC device configuration. (a) Schematic illustration of the workflow of the experimental-computational framework. (b) PSC device configuration.

We fabricated devices based on those input values following the procedure outlined in the Methods and measured their photovoltaic performance, including PCE. We subsequently provided the input-output data to the optimization algorithm, which modeled the problem (i.e., how PCE changes and can be maximized based on input values) and identified promising regions. For this purpose, we employed stable noisy optimization by branch and fit algorithm (SNOBFIT),^75^ which is a well-known DFO algorithm for its ability to construct local surrogate models to make reliable predictions on output and effectively handle noise in data. SNOBFIT has been widely used in optimization of complex chemical reaction systems^78–82^ and demonstrated superior performance compared to several simulation optimization solvers when handling noisy output responses generated in computational experiments.^83^ Using SNOBFIT, we aimed to maximize PCE and identify the optimal device design within the input space without requiring exhaustive trial-and-error across all possible combinations. SNOBFIT follows its algorithmic procedure outlined in Methods to learn from the collected data and generate new sample points for subsequent experimentation. Its predictive models can suggest points that are likely to maximize PCE, or focus on unexplored regions where fewer samples exist, increasing the chances of finding global optima. We changed the device processing conditions based on SNOBFIT’s recommendations and experimentally verified the results by measuring the PCE.

To guarantee the robustness of experiments, we fabricated duplicate devices for each design with each device containing eight independently working pixels, as described in the Methods, to produce up to 24 duplicate pixels for each processing condition. We calculated the median PCE from multiple pixels to represent the output PCE performance. Figure S2 shows an illustration of the distribution of PCE performance obtained from duplicate pixels. We fed the collected data of the median PCE back into the algorithm, creating an iterative process where we would update the data and refine the optimization progress after each experimental batch. The batch-based experimentation provided flexibility to make decisions between batches whether to continue sampling within the same bounds or to adjust the input space when certain regions show significantly lower performance than the rest of the design space. This approach allowed us to integrate our knowledge-based judgment within the optimization process when necessary and concentrate on promising regions.

Using the proposed framework, we initially focused on optimizing the deposition of the perovskite layer, a critical step for achieving high performance. Figure S3 illustrates the deposition process of the perovskite layer, which comprises three steps: a rotation at low speed, followed by a rotation at higher speed, and quenching with an antisolvent to induce film crystallization. In Figure S3, we highlight the two optimized design variables: the low spin rate step of perovskite layer deposition and the antisolvent quenching time, defined as the interval between the spin start and the introduction of the antisolvent, which is timed to occur during the high-speed rotation step. We selected these variables because of their potentially significant impact on the quality of the perovskite film and interface and thereby enhanced overall device performance. In particular, the first step spin rate affects the thickness of the perovskite layer, while the timing of antisolvent introduction influences the film morphology and crystallographic properties. Our reference condition set the lower spin rate to 1,000 rpm and introduced chlorobenzene as the antisolvent 27 s after the start of spin coating.

For the input setup of the SNOBFIT algorithm, we defined the range of lower spin rate as [500, 1,500] rpm and the range of quenching time as [11, 27] seconds. We also set a resolution vector of [1 rpm, 1 s] in SNOBFIT, which ensures that data collection is restricted to points where they can be represented as an integral multiple of the vector, i.e., the allowed spin rate and quenching time were natural numbers between 500 and 1,500 rpm and between 11 and 27 s, respectively.

We present the optimization progress of the two variables in Figures 2a–h. The light gray lines show the subdivision of the search space at each iteration, the blue circle at the top center represents the reference, the green diamonds denote the newly sampled points in each iteration, the dark gray circles indicate the points sampled in previous iterations, and the magenta triangle marks the incumbent solution where the median PCE was the highest among the cumulative data. The magenta lines illustrate the trajectory of the incumbent solution over iterations. Each point in the 2D space corresponds to a PSC design defined by the input values of that point. For the first seven iterations, we employed SNOBFIT’s randomized initial sampling and its size-based search feature that targets the largest subdomains to sample in unexplored regions and collect sufficient data for subsequent model-based search. Additionally, each of iterations 2 through 7 included one model-based sample point, determined from the local surrogate model built around the incumbent solution point. For the last iteration, we set the model-based to size-based search ratio at 0.67 to 0.33, allowing for both local exploitation to refine locally optimal solutions and global exploration to continue the search for global optima.

**Figure 2:**
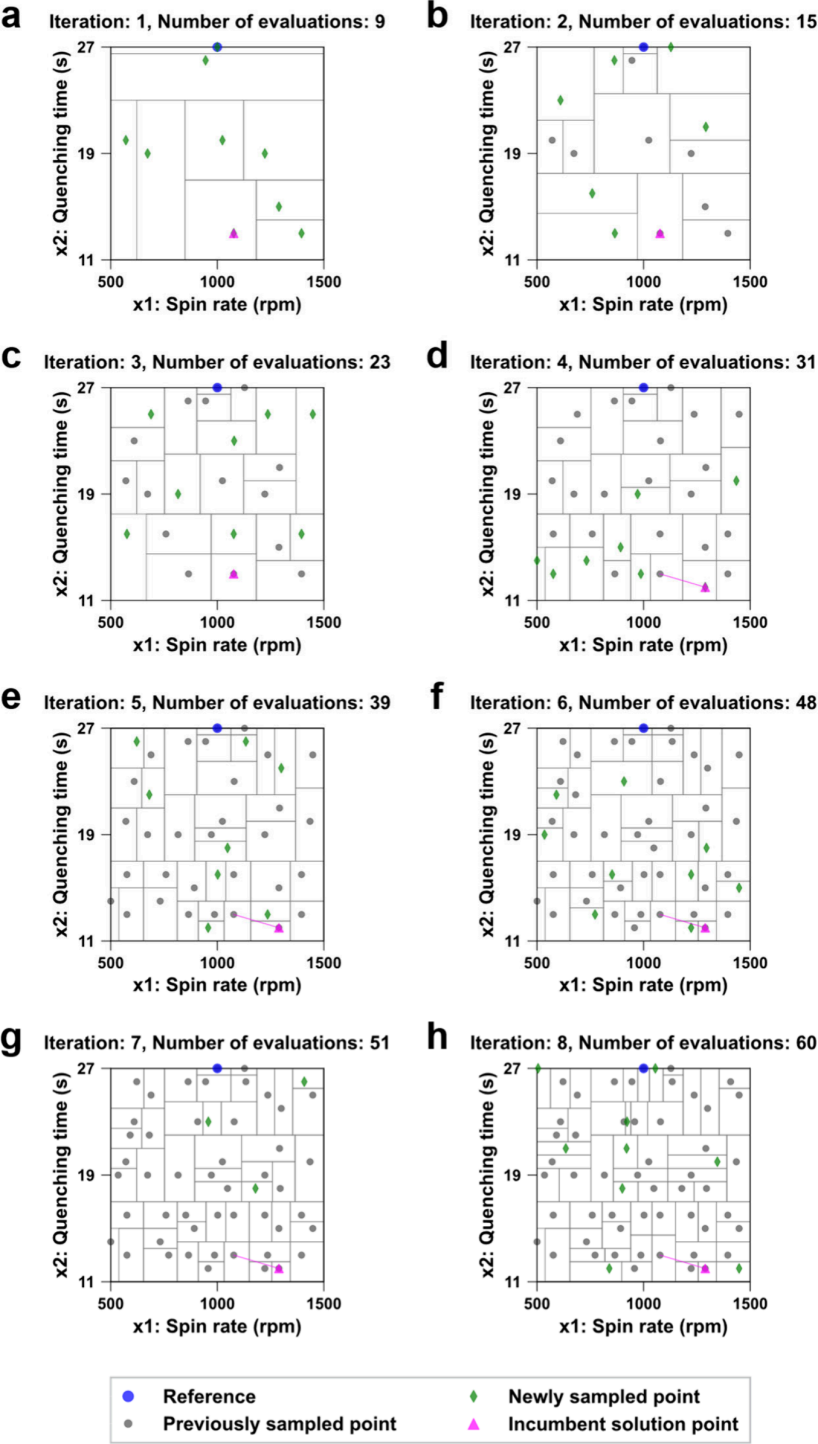
Progress of perovskite layer optimization. (a)–(h) Each panel shows the number of iterations and the cumulative evaluations completed after each iteration. The horizontal axis denotes the spin rate of the first spin step for the perovskite layer deposition, and the vertical axis represents the antisolvent quenching time.

We present the results of the two-dimensional optimization of perovskite layer deposition and compare the best-performing design with the reference in Figure 3. As shown in Figure 3a, the median PCE performance varied across the input space, with no distinct regions of consistently high or low performance, illustrating the nonconvex and noisy nature of the PSC design optimization problem. We identified the best-performing design at (1,289 rpm, 12 s) where the median PCE improved from the reference’s 19.6% to 20.2%, and the maximum PCE of 20.8% surpassed the reference’s 20.3%, depicted in Figure 3b. Other photovoltaic parameters, presented in Figures 3c–e, indicate that the optimized design outperformed the reference in all performance metrics except for the fill factor (FF). We provide the complete device parameters of the reference and best design based on reverse and forward current density–voltage (*J*–*V*) scans as well as maximum power point (MPP) tracking in Figure S4 and Table S1.

**Figure 3:**
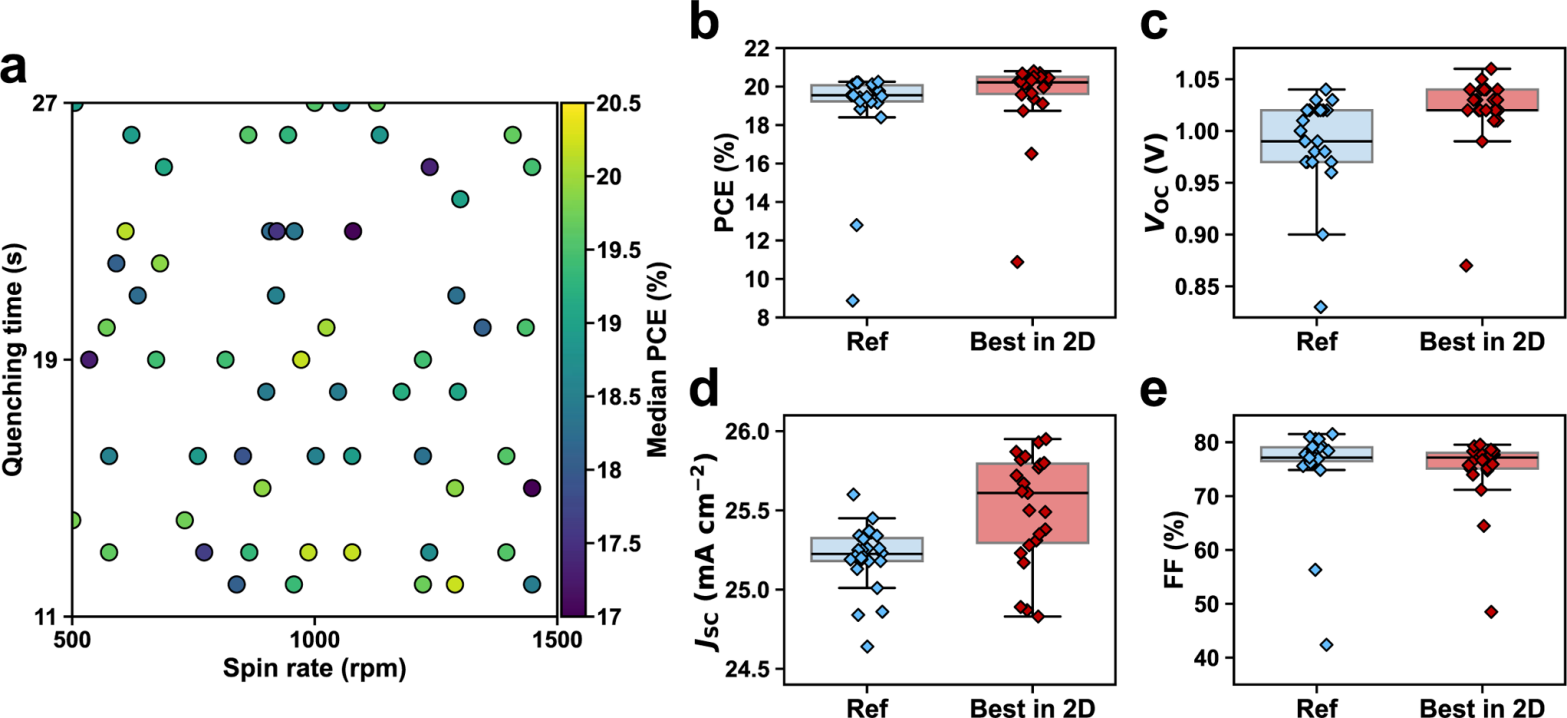
Two-dimensional optimization of the perovskite layer and improved performance. (a) Collected data points in the two-dimensional space and their corresponding median reverse-scanning PCE. (b) PCE, (c) *V*_OC_, (d) *J*_SC_, and (e) FF obtained from reverse *J*–*V* scans.

These results suggest that a high spin rate resulting in a thin perovskite layer as well as early quenching to promote crystallization may be beneficial for the PSCs studied in this work. We look to further analyze these findings through characterization and gain conclusive insights. For instance, Figure S5 presents grazing incidence wide-angle X-ray scattering (GIWAXS) and optical microscopy images of various perovskite films. The GIWAXS images in Figures S5a–c show similar structural features across films with high, medium, and low performance, except for the slight presence of an impurity phase in the medium- and low-efficiency films. The impact on the surface is much more pronounced, as seen in the morphological variations in Figures S5d–f. This difference suggests that the changes made in perovskite deposition primarily have impacted the surface, thereby affecting device performance.

To achieve further improvements, we introduced two additional variables related to the electron transport layer (ETL) into the optimization process: the ethanol-to-titanium precursor mixing ratio in the titanium solution for spray pyrolysis, ranging from 10:1 to 20:1, and the gas flow rate during spray pyrolysis, set at 3, 4, or 5 L min^–1^, as illustrated in Figure S6. These ETL-related variables influence the thickness and uniformity of the film, enabling us to optimize both the perovskite layer and the ETL for improved efficiency. An important benefit of using optimization algorithms in this context is their ability to handle multiple design variables across various layers of PSCs simultaneously, which would be highly challenging to achieve manually due to the variables’ complexity and interconnectedness.

Figure 4 shows the results of the four-dimensional optimization of the perovskite layer and ETL, along with a comparison to the best design achieved from the two-dimensional optimization of the perovskite layer. As depicted in Figure 4a, we sampled a total of 17 points within the defined four-dimensional design space as part of the initial Latin hypercube sampling, which aims to generate a well-distributed set of sample points covering the entire design space. We were able to identify a design with an improved median PCE during this sampling: a spin rate of 1,300 rpm, quenching time of 15 s, an ethanol-to-titanium precursor ratio of 13.5, and a spray rate of 4 L min^–1^ for ETL deposition. The deposition parameters for the perovskite layer, namely, the spin rate and quenching time, that resulted in higher PCE were different than those found in the 2D optimization space. This finding suggests that there is an interdependency among the explored variables and layers. As such, it highlights the importance and promise of leveraging optimization algorithms to address complex interlayer dynamics and to achieve further improvements in device performance. Figure 4b shows that the median PCE improved from 20.2% in the two-dimensional optimization to 21.8% in the four-dimensional optimization. The best-performing cell achieved a maximum PCE of 22.3%. While further data collection is required to continue optimization, these results demonstrate the significant impact of ETL optimization and the potential for even greater improvements by refining the ETL processing variables along with perovskite processing variables.

**Figure 4:**
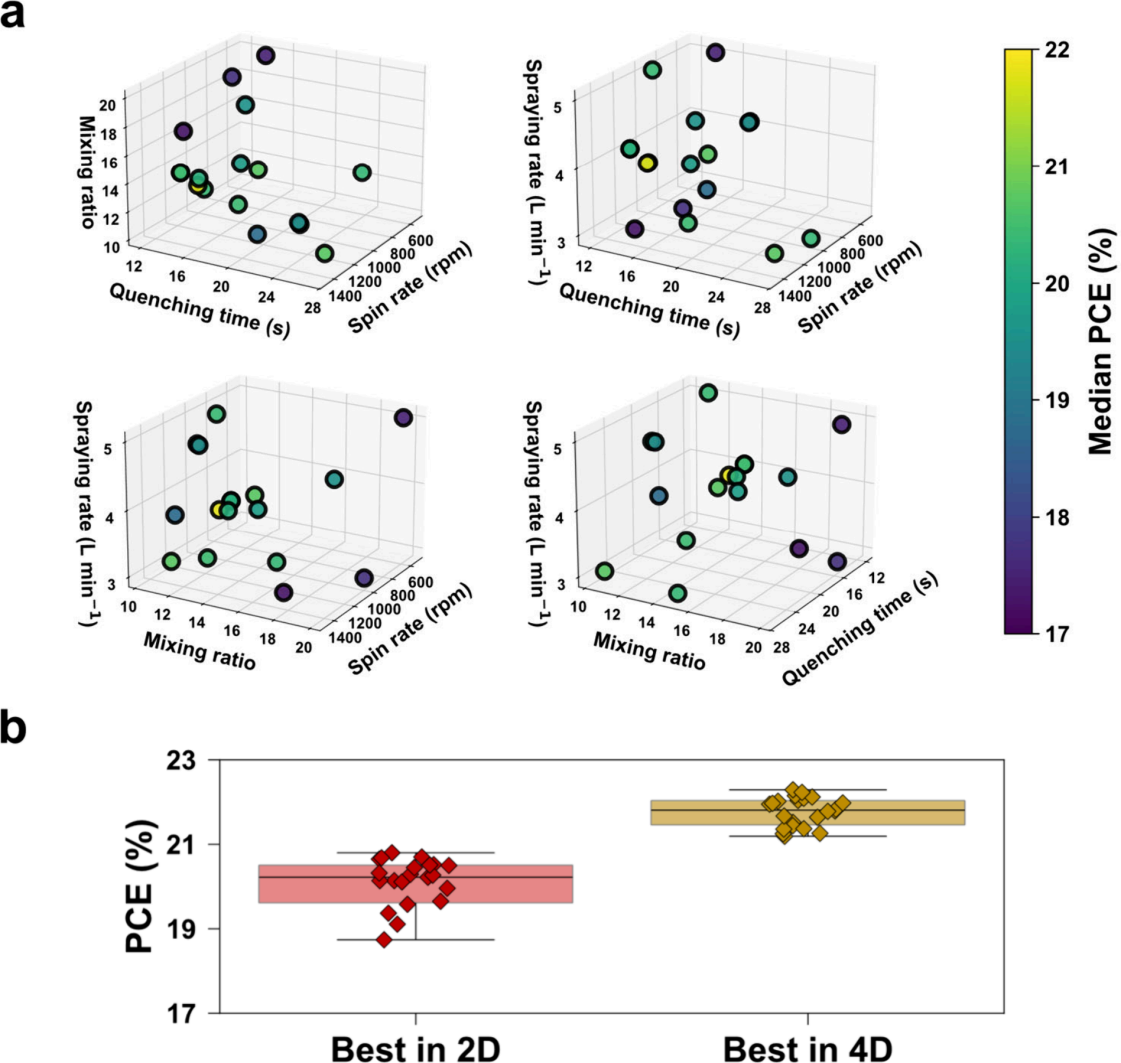
Four-dimensional optimization of perovskite layer and ETL, and improved performance. (a) Collected data points in the four-dimensional space and their median PCE. Each of the four subplots shows a different combination of three variables out of the total of four for illustration. (b) PCE obtained from reverse *J*–*V* scans. The two best designs found from 2D and 4D optimizations are compared against each other.

Another key component of our PSC devices is the hole transport layer (HTL), which facilitates the movement of positive charge carriers from the perovskite layer to the top electrode. For the HTL, we used 2,2′,7,7′-tetrakis­[*N*,*N*-di­(4-methoxy­phenyl)­amino]-9,9′-spirobifluorene mixed with 4-*tert*-butylpyridine, bis­(trifluoroethane)­sulfonimide lithium salt (Li-TFSI), and FK209 Co­(III) TFSI salt as additives, which can increase hole conductivity and reduce phase segregation.^84^ We investigated the impact of two processing variables in the HTL deposition: the concentration of these three additives and the temperature of the HTL precursor solution for mixing and casting. Figure 5a shows the results obtained by increasing or decreasing the concentration of the three additives together by 20% from the reference. The higher PCE achieved with higher additive concentrations indicates that increasing the additive concentrations to enhance conductivity may positively impact the HTL design and overall device performance, which we plan to investigate further. We also observed that raising the temperature of the precursor solution to 75 °C prior to deposition improved the maximum PCE to 22.8%, as shown in Figure 5b, which increased to 23.1% after applying an antireflective coating on the glass substrate side. This result represents the best design and performance achieved in this work. Given the substantial impact of HTL design on device performance, we plan to examine the extent to which these HTL-related variables affect PCE, define the suitable range of each, and integrate them into the systematic optimization problem. Figure 5c and Table 1 summarize the progression of improvements in our design and performance achieved by expanding the search to a six-dimensional space of key variables across various layers in the device structure. The work from two- to six-dimensional optimization required less than 100 tested designs in total.

**Figure 5:**
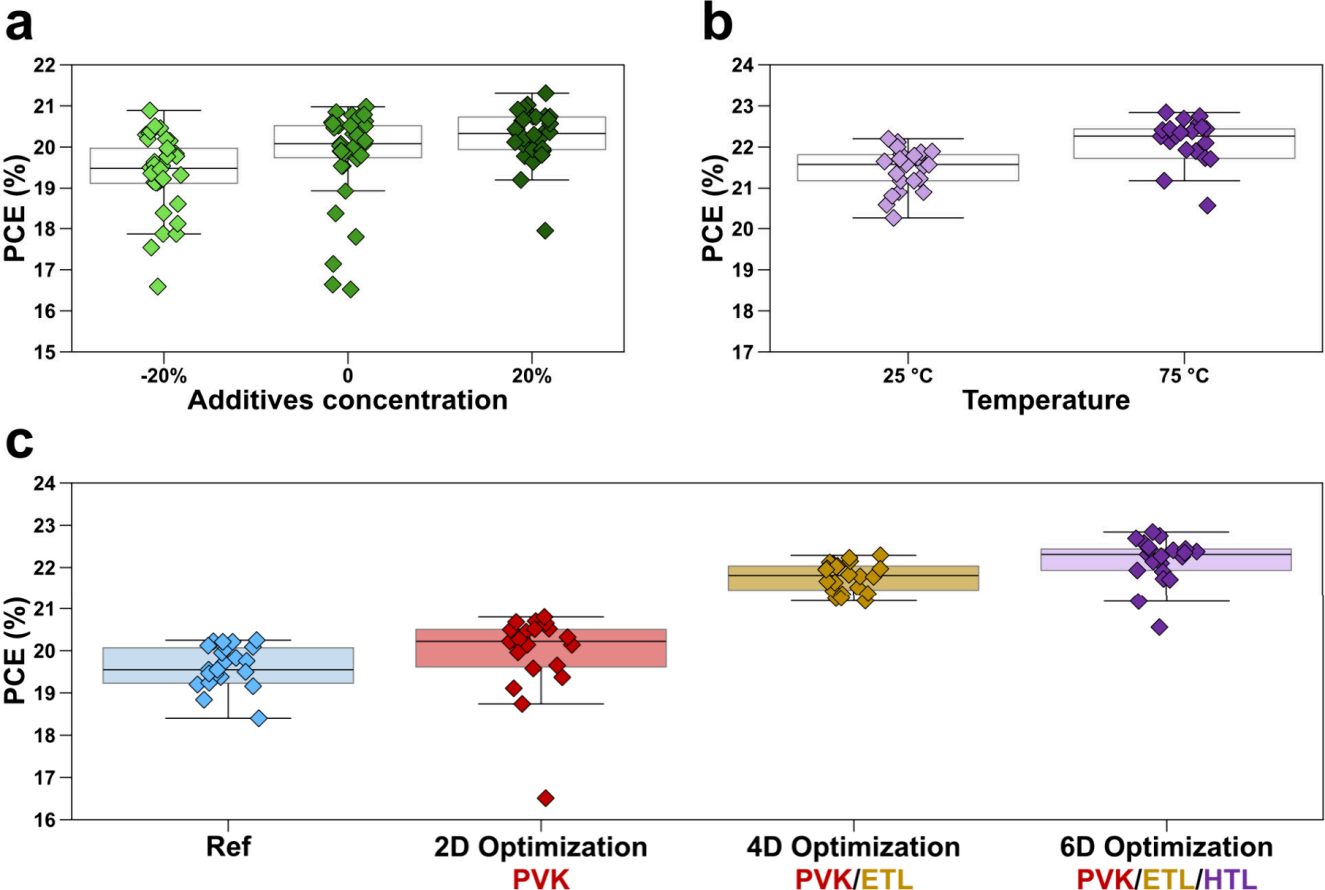
Optimization of HTL variables and overall optimization progress. (a) Reverse-scanning PCE results for different additive concentrations. The horizontal axis represents the additive concentration relative to the reference. (b) Reverse-scanning PCE results for varied temperatures of the HTL precursor solution. (c) Comparison of performance achieved through the optimization of different numbers of variables.

**Table 1:** Top Performance in Each Metric Was Obtained by the Optimization of the Key Design Variables^a^

Degrees of freedom	Layer	Design variables	Reverse-scanning efficiency (%)	*V*_OC_ (V)	*J*_SC_ (mA cm^–2^)	FF (%)	Stabilized efficiency (%)
0	(Unoptimized) reference		20.3	1.04	25.60	81.5	19.1
2	PVK	Spin rate of spin-coating deposition	20.8	1.06	25.95	79.6	19.7
		Antisolvent introduction timing					
4	PVK	Spin rate of spin-coating deposition	22.3	1.10	25.98	83.5	22.0
		Antisolvent introduction timing					
	ETL	Precursor concentration					
		Flow rate of spraying deposition					
6	PVK	spin rate of spin-coating deposition	22.8 (23.1)	1.12 (1.11)	26.61 (27.15)	81.4 (81.3)	22.5 (23.1)
		Antisolvent introduction timing					
	ETL	Precursor concentration					
		Flow rate of spraying deposition					
	HTL	Additives concentration					
		Temperature of precursor solution					

a PVK, ETL, and HTL refer to the perovskite layer, electron transport layer, and hole transport layer, respectively. Values in parentheses correspond to the performance achieved with the application of an antireflective coating.

In summary, we systematically improved the performance of PSCs through algorithm-guided experimentation. With fewer than 100 tested designs, we optimized six design variables across the fully assembled device architecture and achieved a substantial increase in PCE from the reference’s maximum of 20.3% to the best design’s 22.8% without an antireflective coating and 23.1% with the coating. This rapid advancement highlights not only the effectiveness of the optimization approach but also the significant impact of the numerous variables in PSC fabrication, reinforcing the importance of adopting a systematic approach for optimization. Future work will aim to further enhance device performance by optimizing with more degrees of freedom and use advanced characterization techniques to study the identified optimal and suboptimal designs. Furthermore, we intend to integrate ML approaches to analyze the collected data after optimization, deriving insights from a data-driven perspective. Our framework is independent of specific techniques or manufacturers and can be readily extended to other deposition methods. Moreover, the framework serves as a systematic tool for addressing intricately interconnected variables in science and engineering problems that are challenging to optimize manually.

## Supplementary Material



## Notes

Experimental methods, fabrication schematics, supporting figures and a table summarizing device optimization and characterization results (PDF)
