# Supplementary material for: Algorithm-Guided Experimentation for Optimization of High-Performance Perovskite Solar Cells
Source: ACS Energy Lett. 2025 Nov 10;10(12):6190–9. doi: 10.1021/acsenergylett.5c02477 (PMC12706828; doi:10.1021/acsenergylett.5c02477)
Supplement: Supplementary file 1 [file nz5c02477_si_001.pdf]

Supporting Information

Algorithm-Guided Experimentation for Optimization of High-Performance Perovskite Solar Cells

Donghyun Oh[1], Sanggyun Kim[2], Carlo A. R. Perini[2],
Juan-Pablo Correa-Baena[2,3] & Nikolaos V. Sahinidis[4,1]**

[1]School of Chemical and Biomolecular Engineering, Georgia Institute of Technology, Atlanta, GA 30332, USA

[2]School of Materials Science and Engineering, Georgia Institute of Technology, Atlanta, GA 30332, USA

[3]School of Chemistry and Biochemistry, Georgia Institute of Technology, Atlanta, GA 30332, USA

[4]H. Milton Stewart School of Industrial and Systems Engineering, Georgia Institute of Technology, Atlanta, GA 30332, USA

*Corresponding Authors: Juan-Pablo Correa-Baena, jpcorrea@gatech.edu;
Nikolaos V. Sahinidis, nikos@gatech.edu

Materials and Methods

Materials

Cesium iodide (CsI) and lead iodide (PbI_2, 99.99%) were purchased from TCI. Formamidinium iodide (FAI) was purchased from Greatcell Solar Materials. Phenethylammonium iodide (PEAI) was purchased from Dyenamo. 2,2',7,7'-tetrakis[N,N-di(4-methoxyphenyl)amino]-9,9'-spirobifluorene (Spiro-OMeTAD) was purchased from 1-Materials. Bis(trifluoroethane)sulfonimide lithium salt (Li-TFSI), FK209 Co(III) TFSI salt, titania paste, and titanium diisopropoxide bis(acetylacetonate) (75 wt. % in isopropyl alcohol) were purchased from Sigma-Aldrich. Acetonitrile (ACN, 99.8%), chlorobenzene (CB, 99.5% for perovskite quenching and 99.9% for Spiro-OMeTAD), dimethyl sulfoxide (DMSO, ≥ 99.9%), ethanol (≥ 99.5%), 4-tert-butylpyridine (TBP), isopropyl alcohol (IPA, anhydrous), and N,N-Dimethylformamide (DMF, 99.8%) were purchased from Sigma-Aldrich. Acetone (≥ 99.5%) and IPA used for substrate cleaning were purchased from VWR Chemicals. Gold pellets (Au, 99.999%) were purchased from Kurt J. Lesker.

Fabrication of perovskite solar cells

Perokvsite solar cells (PSCs) were fabricated with the following structure: glass/fluorine-doped tin oxide (FTO)/c-TiO_2/mp-TiO_2/PEAI/$Cs_{0.09}FA_{0.91}PbI_3$/PEAI/Spiro-OMeTAD/Au. Patterned FTO glass substrates were sequentially sonicated for 15 minutes each in 2% mucasol (schülke) solution and deionized water, then for 10 minutes each in acetone and IPA, and were subsequently dried with nitrogen. The cleaned FTO glasses underwent additional cleaning via a 15-minute ultraviolet/ozone (UVO) treatment to remove organic contaminants and improve surface wettability. The compact TiO_2 layer was then deposited through spray pyrolysis from a 11.52 mL

solution of a mixture of ethanol, acetonitrile, and titanium diisopropoxide bis(acetylacetonate). The deposition was made over several cycles, each consisting of a 15-second spraying phase followed by a 30-second intermission. The flow rate of spraying gun was set at 3-5 L min^{-1}. The substrates were annealed at 450°C for the duration of this process, including a 30-minute post annealing period. The mesoporous TiO_2 layer was deposited on top of the compact TiO_2 layer by spin coating at 4,000 rpm for 10 seconds, using 60 μL of a 150 mg mL^{-1} solution of titania paste dissolved in ethanol. The mesoporous TiO_2 layer was then dried on a hot plate at 100°C for a minimum of 10 minutes and subsequently sintered at elevated temperatures, the maximum being 450°C. The TiO_2 film underwent an additional UVO treatment for 15 minutes, with the exception of the HTL optimization. followed by passivation layer deposition using 90 μL of 1 mg mL^{-1} solution of PEAI in IPA via spin coating at 5,000 rpm for 20 seconds. 1.2M Cesium formamidinium lead iodide ($Cs_{0.09}FA_{0.91}PbI_3$) perovskite film with 5% excess Pb was subsequently deposited via spin coating a 90 μL of perovskite precursor solution dissolved in a mixed solvent (DMF:DMSO = 2:1 v/v). The spin coating process for perovskite involved two steps: initially at a relatively lower spin rate, such as 1,000 rpm for 10 seconds for the reference devices, and subsequently at a higher rate of 6,000 rpm for 20 seconds. During the second step, 250 μL of chlorobenzene (CB) was dispensed onto the substrate, working as the antisolvent. The substrates were annealed at 150°C for 10 minutes after perovskite deposition. The previously described PEAI passivation was also applied on top of the perovskite layer. Then the hole transport layer was deposited by spin-coating a 90 uL of Spiro-OMeTAD solution at 3,000 rpm for 30 seconds. The Spiro-OMeTAD solution was prepared before the deposition by mixing it with CB, TBP, Li-TFSI salt solution (1.8 M in ACN), and Co-TFSI salt solution (0.25 M in ACN), and was mixed for at least 30 minutes before deposition. After the hole transport layer deposition, a gold layer with a thickness of 45-50 nm was

evaporated on top as the metal electrode, using a shadow mask to create eight independent working pixels on a single substrate. For the optimization of the perovskite layer and electron transport layer, we fabricated three duplicate devices for each design, which gave us up to 24 pixels for each design except when some pixels were non-functional due to issues such as non-uniform film coverage. For hole transport layer optimization, we fabricated four duplicate devices for each design, yielding up to 32 pixels to enhance the reliability of the results.

Photovoltaic performance measurement

The photovoltaic parameters of PSC were extracted from *J*–*V* curves measured with a Fluxim solar simulator, operating under AM1.5G illumination and a scan rate of 50 mV s^{-1}. Both forward and reverse scans were conducted to obtain *J*–*V* curves, and the stabilized PCE was determined based on maximum power point (MPP) tracking performed over a 2-minute period. Each PSC device consisted of 8 working pixels and the *J*–*V* curve was measured for each pixel to extract key photovoltaic parameters, including V_{OC}, J_{SC}, FF, and PCE.

Stable Noisy Optimization by Branch and Fit

Stable Noisy Optimization by Branch and Fit (SNOBFIT) is a model-based derivative-free optimization (DFO) algorithm that follows a branch-and-fit scheme to subdivide the input domain into several sub-domains and build a local quadratic model for each point and its nearest neighbors. In each iteration of optimization, SNOBFIT generates sampling points based on model predictions to locate local optima while also sampling unexplored regions to increase the chances of finding global optima. It iteratively updates the input domain partitioning and surrogate models as new data is provided. Unlike many other solvers, SNOBFIT allows users to provide the expected accuracy of each data observation, enabling it to adjust its model to fit closer to accurately

measured observations than noisy observations, thereby building reliable surrogate models even in the presence of noise in data.

Supporting Figures

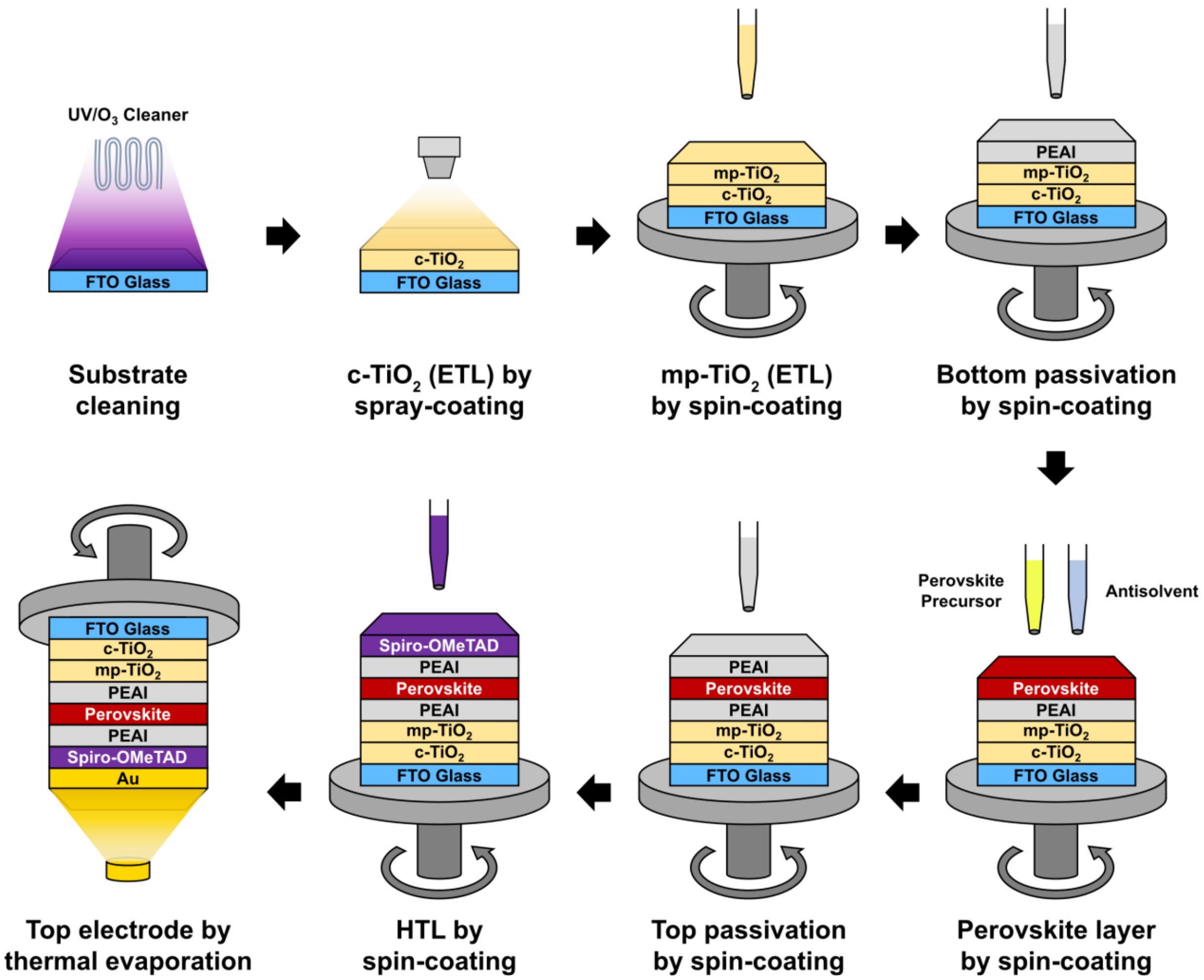

Figure S1. Schematic illustration of the fabrication process. ETL and HTL denote the electron transport layer and hole transport layer, respectively.

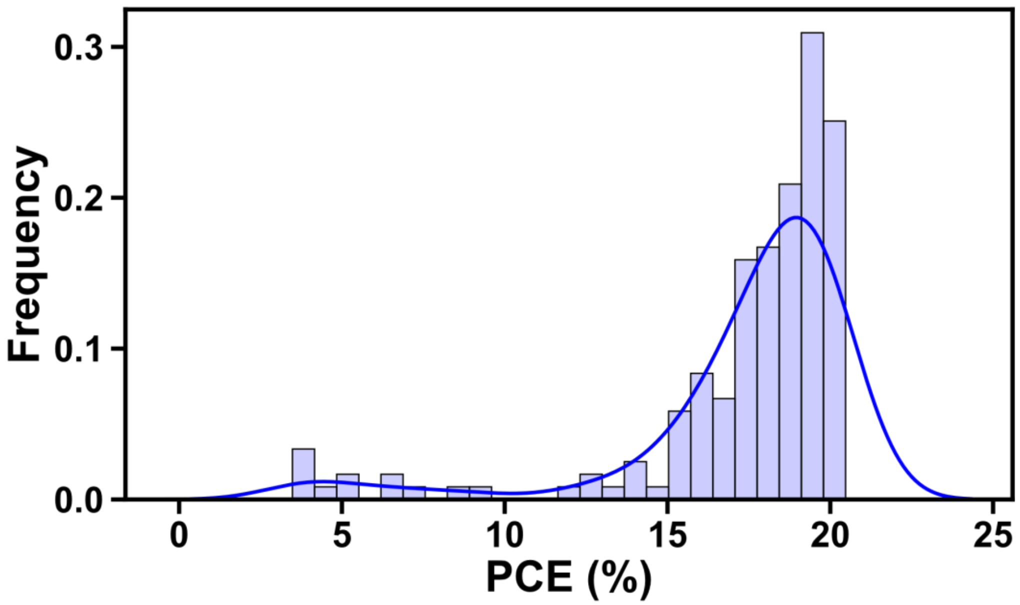

Figure S2. Illustration of the non-normal distribution of PCE performance across multiple pixels fabricated under identical processing conditions.

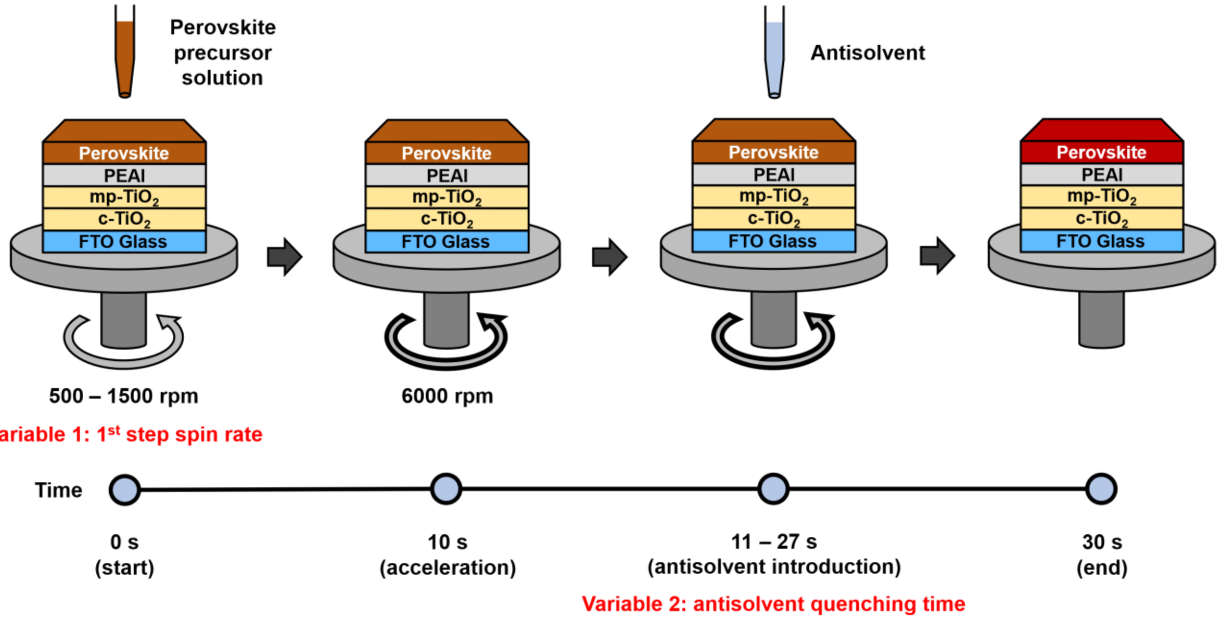

Figure S3. Schematic illustration of perovskite layer deposition.

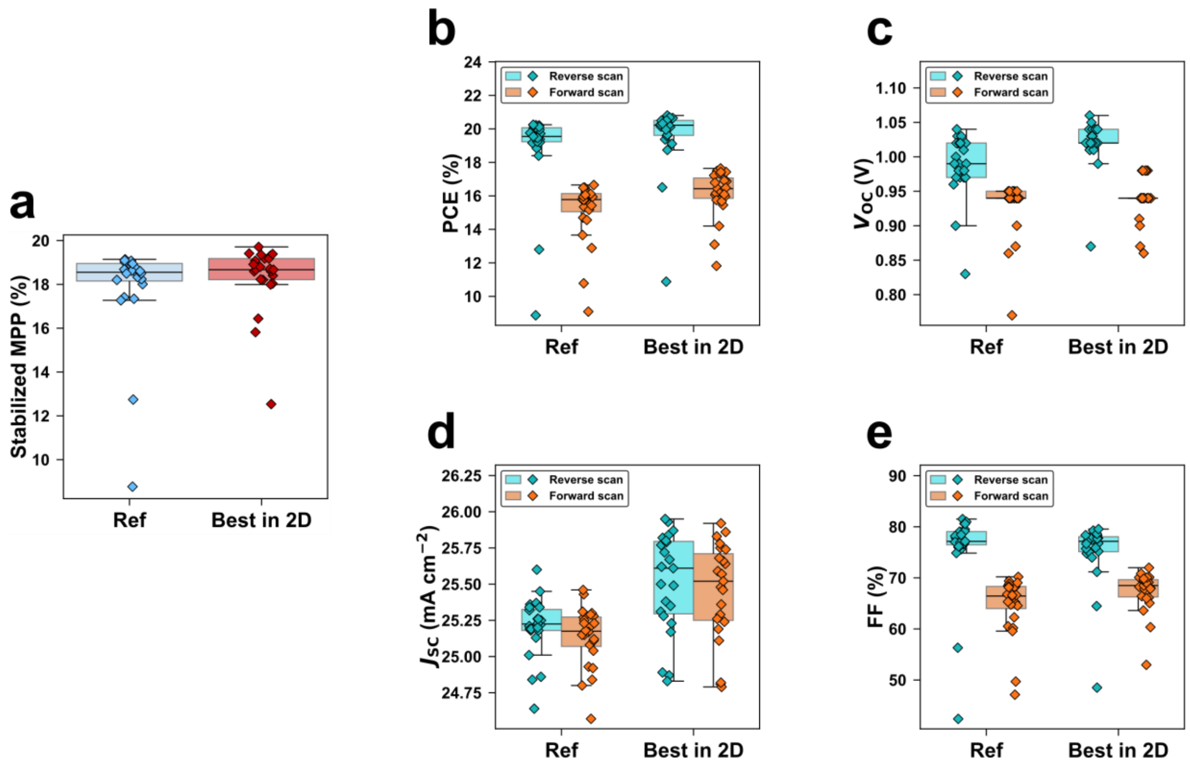

Figure S4. Device parameters of reference and best obtained from two-dimensional optimization of perovskite layer. (a) Stabilized efficiency obtained after two minutes of maximum power point tracking. (b) PCE, (c) V_{OC}, (d) J_{SC}, and (e) FF obtained from reverse and forward J–V scans.

Table S1. Summarized device parameters of reference and best obtained from two-dimensional optimization of perovskite layer deposition. Values outside the parentheses correspond to the maximum, while those in parentheses represent the median.

Variation	Reverse-scanning efficiency (%)	V_{OC} (V)	J_{SC} (mA cm^{-2})	FF (%)	Stabilized efficiency (%)
Ref	20.3 (19.6)	1.04 (0.99)	25.60 (25.22)	81.5 (77.2)	19.1 (18.6)
Best in 2D	20.8 (20.2)	1.06 (1.02)	25.95 (25.61)	79.6 (77.2)	19.7 (18.7)

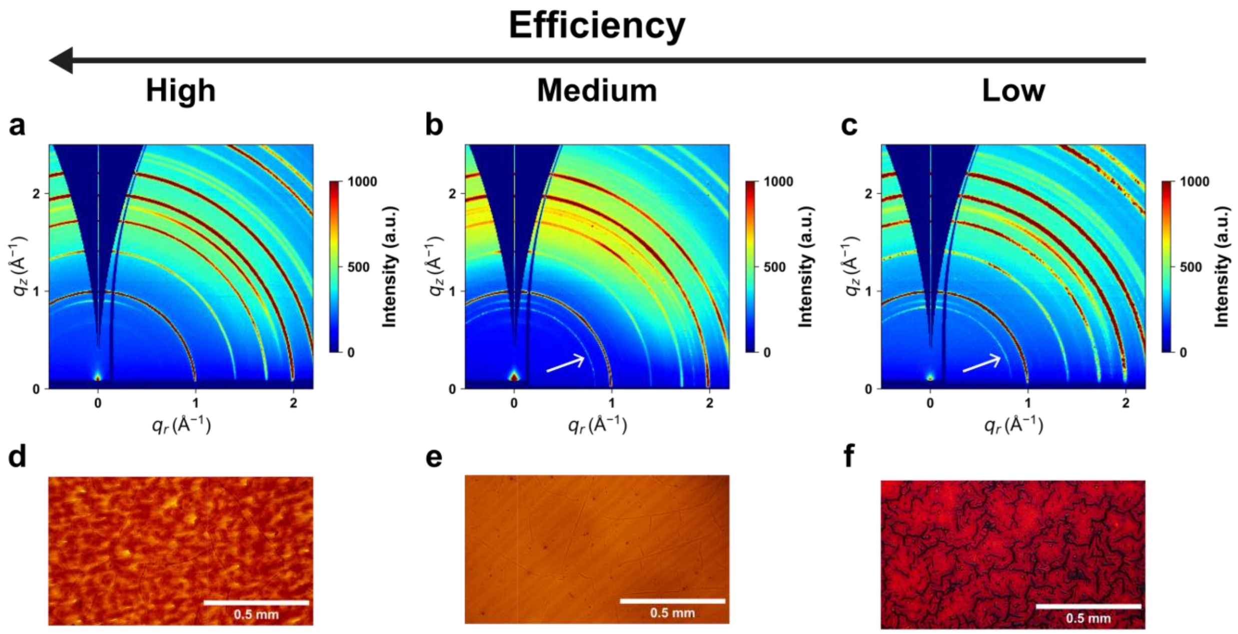

Figure S5. GIWAXS images of perovskite films with (a) high, (b) medium, and (c) low efficiency, and optical microscopy images of perovskite films with (d) high, (e) medium, and (f) low efficiency. The white arrows in the GIWAXS images point to the observed impurity phase. Perovskite films for both GIWAXS and microscopy were prepared on top of $FTO/TiO_2/PEAI$.

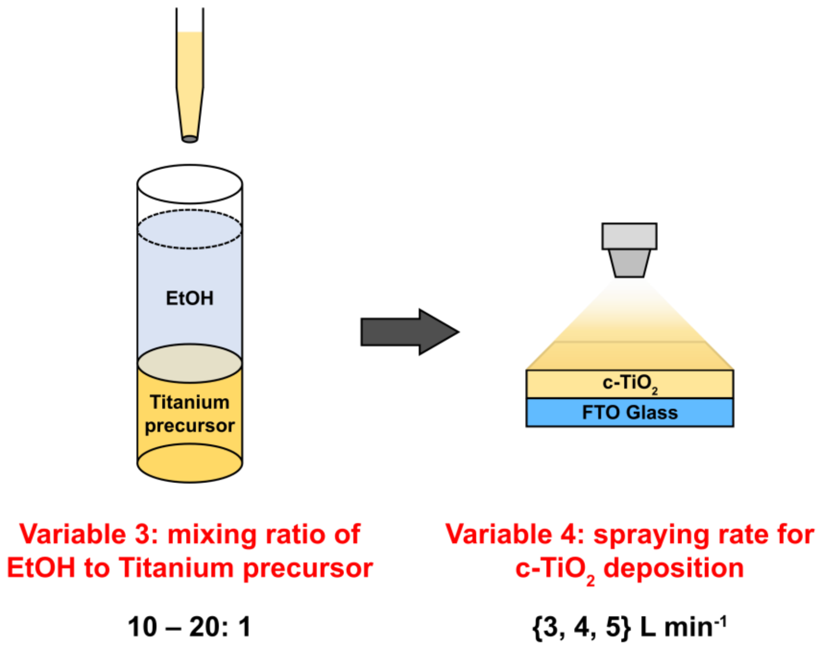

Figure S6. Schematic illustration of ETL deposition and the two related design variables.
